# How Self-Employment Influences Three Forms of Income in Later Life: A Swedish Registry Data Study

**DOI:** 10.1093/geroni/igaf122.443

**Published:** 2025-12-31

**Authors:** Cal Halvorsen, Melody Almroth, Kuan-Yu Pan, Alicia Nevriana, Jessie Gevaert, Daniel Falkstedt

**Affiliations:** Washington University in St. Louis, St. Louis, Missouri, United States; Karolinska Institutet, Solna, Stockholms Lan, Sweden; Karolinska Institutet, Solna, Stockholms Lan, Sweden; Karolinska Institutet, Solna, Stockholms Lan, Sweden; Vrije Universiteit Brussel, Brussel, Brussels Hoofdstedelijk Gewest, Belgium; Karolinska Institutet, Solna, Stockholms Lan, Sweden

## Abstract

Research that considers the relationship between self-employment and income has generated mixed results, possibly due to selection bias and imprecise measurements of self-employment. In response, we aimed to estimate the treatment effects of self-employment on three types of income—income from work, adjusted household income, and receipt of social benefits—while controlling for selection into self-employment, specifying self-employment types, and identifying treatment effects by gender. We used data from the Swedish Work, Illness, and Labor Market Participation cohort (born 1959-1969; ∼1.2 million individuals). To reduce selection bias, we created entropy balancing weights to balance the self-employed and wage/salary employment groups in 2014 on sociodemographic, work, and health factors from prior years. We included these weights in regression models to predict different types of income in 2019. Compared to wage/salary employment and controlling for multiple job holding, we estimate that self-employment in 2014 led to lower annual work income (-213,777 SEK, p<.001; ∼$21,000) and adjusted household income (-55,831 SEK, p<.001; ∼$5,500) in 2019, with sole proprietors and women being the most financially vulnerable. Although self-employment in 2014 was negatively related to receipt of social benefit income in 2019 (OR=.729, p=.002), this effect was lost once accounting for multiple job holding. These results show how self-employment’s influence on income is multifaceted. Future research should investigate the motivations for pursuing self-employment past midlife and their interactions with gender and household dynamics, given that it may be a useful form of work for people needing flexibility (e.g., caregivers) and desiring more autonomy.

